# Systematic Review of Literature on Single-Nucleotide Polymorphisms Within the Oxytocin and Vasopressin Receptor Genes in the Development of Social Cognition Dysfunctions in Individuals Suffering From Autism Spectrum Disorder

**DOI:** 10.3389/fpsyt.2019.00380

**Published:** 2019-05-31

**Authors:** Krzysztof Maria Wilczyński, Andrzej Siwiec, Małgorzata Janas-Kozik

**Affiliations:** ^1^Pediatric Centre of John Paul II in Sosnowiec Sp. z o.o., Sosnowiec, Poland; ^2^Department of Psychiatry and Psychotherapy of Developmental Age, Medical University of Silesia, Katowice, Poland

**Keywords:** oxytocin receptor, arginine vasopressin receptor, autism spectrum disorder (ASD), social cognition, oxytocin, vasopressin, systematic review

## Abstract

**Introduction:** Autism spectrum disorder (ASD) is found in virtually all population groups regardless of ethnic or socioeconomic backgrounds. Among others, dominant symptoms of autism persistent throughout its course of development include, inter alia, qualitative disorders of social communication and social interactions. Numerous studies have been performed on animal models as well as groups of healthy individuals to assess the potential role of oxytocinergic and vasopresynergic systems in normal social functioning. These studies have also discussed their potential participation in the development of social cognition dysfunctions in the course of ASD. This literature review aimed to identify studies examining single-nucleotide polymorphisms of the oxytocin (OXT) and arginine vasopressin (AVP) receptor genes and their differential effects on social cognitive dysfunction in the development of ASD.

**Methods:** A systematic review of literature published within the last 10 years and accessible in PubMed, Google Scholar, Cochrane Library, and APA PsycNET databases was conducted by each author separately. Inclusion criteria required that articles should 1) be published between January 2008 and August 2018; 2) be published in English or Polish; 3) be located in periodical publications; 4) focus on the role of polymorphisms within oxytocin and vasopressin receptor genes in autistic population; 5) provide a clear presentation of the applied methodology; and 6) apply proper methodology.

**Results:** From the 491 studies qualified to the initial abstract analysis, 15 met the six inclusion criteria and were included in the full-text review.

**Conclusions:** The analysis of available literature seems to indicate that there is an association between social cognition dysfunctions in the course of autism and selected alleles of polymorphisms within the OXT receptor AVP 1A receptor genes. However, previous studies neither specify the nature of this association in an unequivocal way nor select genotypes that are the basis for this association.

## Introduction

Autism spectrum disorder (ASD; also autism) is found in virtually all population groups regardless of ethnic or socioeconomic background (1). Dominant symptoms of autism persistent throughout the course of its development include qualitative disorders of social communication and social interactions, deficits in social reciprocity, and/or excessive adherence to rituals and fixed patterns of behavior (2). These symptoms are observed permanently regardless of the person’s background or situation (2, 3). The first symptoms of ASD usually occur in early childhood; however, symptoms may remain undetected until environmental challenges surpass the social abilities of the autistic individual to deal with them. Communication and social functioning disorders observed in the course of ASD are linked with so-called social cognition (SC) deficits. SC refers to all processes necessary for the identification, interpretation, and sending of a behavioral response to information, which reaches the central nervous system (CNS) and which concerns social functioning. Such functions include the ability to process emotions, social perception, and attribution of mental states to self and others in order to interpret and anticipate behaviors (4, 5). These abilities are essential to normal functioning in society, and over the long term, their deficits may lead to the individual’s exclusion and stigmatization (6).

Based on interactions between genetic and environmental factors, it is believed that the etiology of ASD is multifactorial. Studies of uniovular twins have revealed the presence of ASD in both siblings in 70–90% of cases (7, 8). This suggests that genetic factors may play a key role in the pathogenesis of at least some ASD cases (9). Current literature provides information on hundreds of genes and their variants that seem to be linked with ASD. In a meta-analysis published in 2015 by Warrier et al. (10), the authors outlined literature reporting 552 different genes that were suspected to be associated with ASD, while according to a review by Tsur et al. published in 2016, there are currently 651 genes associated with ASD, out of which 30 are strongly supported by literature (11).

Recently, specific interest has been paid to the so-called single-nucleotide polymorphisms (SNPs) (12). These are loci within a genome, which include a single base pair and which have two or more alleles, each with appreciable frequency within a population. Their presence in coding sequences of genes may be a reason for alternations in the expression level or the structure of the coded protein and therefore may lead to its dysfunction.

Oxytocin (OXT) and arginine vasopressin (AVP) are neuropeptides synthesized in the hypothalamus and are closely related to each other both genetically and structurally (13, 14). They belong to the family of nonapeptides, and their lineage can be traced to invertebrates (15, 16). Homologs of these neuropeptides are believed to have existed for at least 700 million years (17) and are present in selected invertebrates and virtually all vertebrates (15). OXT, AVP, and their homologs vary by a single amino acid for OXT and two for AVP. Vertebrates usually have two homologs, while invertebrates usually have one (17). OXT and AVP may both act *via* synaptic signaling within specific pathways or act as neurohormones binding to receptors distant from the place of secretion (17). Across all species and different variants of these nonapeptides and their receptors, the following three core characteristics seem to be evolutionary conserved (15):

1. place of secretion—mainly the neurosecretory regions of the CNS (17);
2. are influenced by gonadal steroids and their function is sexually dimorphic; and
3. are crucial for facilitating social and reproductive behaviors; and although their role in these functions is highly evolutionarily conserved, the specific mechanisms of their function are quite diverse (17)

In humans, OXT and AVP exert their actions peripherally (after secretion by neurohypophysis) and in the CNS where they are considered to be at the core of pathways responsible for both social interactions and reproduction. OXT and AVP are neuromodulators, capable of, inter alia, shaping the development of the human brain and increasing social reactivity and resistance to stressors. Due to the strong influence of OXT pathways on the development of the human neocortex, nonapeptides are considered to be crucial for its evolution and therefore a prerequisite for the appearance of SC and verbal communication (14).

Initial descriptions of OXT centered on its role in uterine contractions and modulation of lactation (13). Further research demonstrated that OXT was responsible for, among others, protecting a baby’s brain from hypoxia during delivery, modulating the development of the neocortex, increasing resistance to stressors, and influencing social reactivity (14). As far as AVP, it was first described for its central role in water homeostasis by its regulation of kidney functions (13). Further studies linked it to functions such as defensive behaviors and modulation of social bonds (14). OXT and vasopressin’s intricately similar characteristics, ability to cross-bind to each other’s receptors, and supposed common evolutionary ancestry inspired some hope for better answers (18, 19). Numerous studies on animal models and groups of healthy individuals point to the potential role of oxytocinergic and vasopresynergic systems in normal social functioning (14, 20).

The literature on social functioning and OXT receptor (OXTR) polymorphisms in various populations is quite extensive and seems to point toward probable link between them. For example in their 2012 study, Chen and Johnson showed that in a group of 178 healthy individuals, the “A” allele of SNP rs2254298 in the OXTR gene is associated with attachment anxiety in females and autism traits in males (21). Similar results were obtained by Rijlaarsdam et al. in a 2017 study on the factors contributing to the development of autistic traits in children (22). They demonstrated that higher methylation levels of the OXTR gene were associated with social deficits in patients who were carriers of two “G” alleles of SNP rs53576 in the OXTR gene. A 2016 study by McDonald et al. showed that SNPs rs53576 and rs2254298 in the OXTR gene were associated with levels of empathy at 24 and 30 months as well as quality of parent–child interactions at 15 and 18 months (23). The association between AVP and SC is based primarily on animal models, due to limited literature on human subjects. However, for example, a 2007 study by Prichard et al. showed that SNPs in arginine vasopressin 1A receptor gene (AVPR1a) were associated with reproductive behaviors in humans (24).

**Figure 1:**
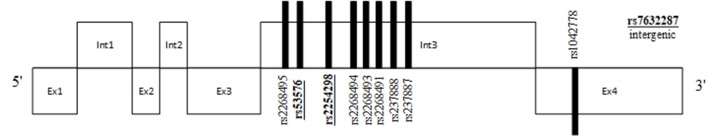
Position of commonly studied single-nucleotide polymorphisms (SNPs) and their relation to exons and introns of the oxytocin receptor (OXTR) gene. SNPs in bold and underlined were found to be associated with social cognition deficits.

**Figure 2:**
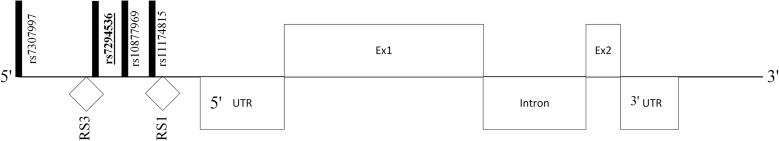
Position of commonly studied SNPs and their relation to exons, introns, and microsatellites (RS1 and RS3) of the arginine-vasopressin 1A receptor gene (AVPR1a) gene. SNPs in bold and underlined were found to be associated with social cognition deficits.

In a meta-analysis published in 2015 by Warrier et al., the authors reviewed literature for 552 genes that were suggested to be associated with ASD (10). Among variants that demonstrated a statistically significant effect was the “G” allele of SNP rs237887 in the OXTR gene [mean odds ratio (OR) = 1.163 (1.002–1.349); *p* = 0.047]. This outcome was also replicated in a meta-analysis of different SNPs in the OXTR gene, conducted by LoParo and Waldman in 2015 (25). LoParo and Waldman showed that aside from rs237887, also SNPs rs2254298, rs2268491, and rs7632287 were found to be significantly associated with ASD. Further studies have also discussed the potential participation of oxytocinergic and vasopresynergic systems in the development of SC dysfunctions in the course of neurodevelopmental disorders (13). This led to the emergence of a number of studies focused on the effects of intranasal application of OXT and AVP on SC in both neurotypical and ASD populations. However, in a paper published in 2019 by Wang et al., the authors conducted a systematic review and meta-analysis of these studies and pointed out that intranasal administration of OXT, in comparison with placebo, presented no significant effect on core symptoms of ASD [social function: standardized mean difference (SMD) = 0.03; 95% confidence interval (CI): −0.19 to 0.25; *p* = 0.7; repetitive behaviors: SMD = 0.01; 95% CI: −0.26 to 0.27; *p* = 0.9]. The overall heterogeneity of analyzed studies was below 50% and equaled *I* ^2^ = 46.4% (*p* = 0.025) for social functioning and *I* ^2^ = 37% (*p* = 0.12) for repetitive behaviors (26). OXT and AVP receptors are G-protein-coupled receptors consisting of seven transmembrane domains. For AVPR, there are at least three known subtypes of this receptor, which have been identified (V_1_A, V_1_B, and V_2_); however, most research in SC has focused on AVPR1a due to its expression in the CNS. The OXTR gene is located at the 3p25-3p26.2 locus, while the AVPR1a gene is located at 12q14-15 and is characterized by three microsatellites located in the 5′ flanking region (27, 28). Locations of the most common SNPs in studies associating their correlation with ASD and SC are depicted in **Figures 1** and **2**. This literature review aimed to identify and summarize studies examining SNPs of the OXTR and AVPR genes and their differential effects on social cognitive dysfunction in the course of ASD that were published between January 2008 and August 2018.

## Methods

This review of literature focused on papers published in the last 10 years, located *via* MEDLINE/PubMed, Cochrane Library, and APA PsycNET as well as the Google Scholar browser. The following keywords were used: “autism spectrum disorder,” “ASD,” “autism,” “social deficits,” “social cognition,” “social affect,” “oxytocin receptor gene,” “oxytocin,” “oxytocin receptor,” “OXTR,” “polymorphism,” “vasopressin,” “arginine vasopressin,” “vasopressin receptor,” “arginine vasopressin receptor gene,” “arginine vasopressin receptor,” and “AVPR.”

The analysis included Polish and English language texts published in recognized journals, which were original papers or reviews/meta-analyses. The texts were browsed by each author individually and then underwent a three-stage selection process. The first stage, based on the title and a preliminary analysis of the abstract, investigated if the publication concerned the subject of the review. During the second stage, the abstract was analyzed in terms of the studied parameters, characteristics of the studied group and control group, and methodology. Papers with substantial methodological errors were excluded (e.g., a lack of proper verification of ASD diagnosis) together with those which were close but not in line with the subject of interest, as well as texts with no clinical group of a confirmed ASD diagnosis. At the third stage, a preliminary analysis of full texts of publications was carried out. Inclusion criteria required that articles should 1) be published between January 2008 and August 2018; 2) be published in English or Polish; 3) be located in periodical publications; 4) focus on the role of polymorphisms within OXTR and AVPR genes in the ASD population; 5) provide a clear presentation of the applied methodology (i.e., exclusion/inclusion criteria), demography of participants, and methods used to confirm the diagnosis; and 6) apply good methodology (i.e., reliable instruments, validated for the target population; clearly stated research question; and satisfactory description of statistical methods and clear presentation of outcomes).

## Results

**Figure 3:**
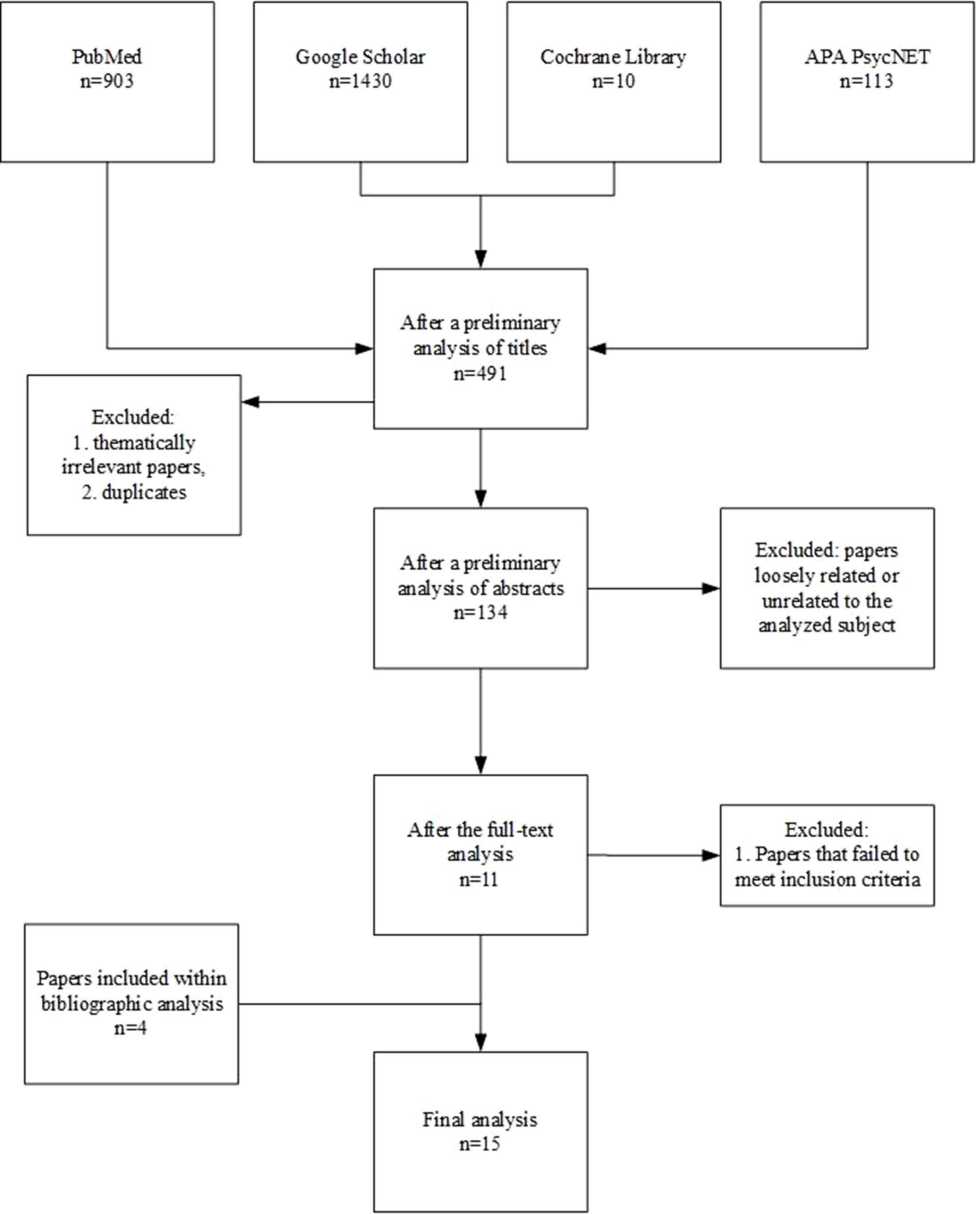
Papers’ qualification procedure for the review.

The initial screening of the literature identified 2,456 publications. After thematically irrelevant papers and duplicates were excluded, 491 studies were included for further analysis. During the preliminary analyses of abstracts, 357 papers were excluded due to a loose relation to the analyzed subject (e.g., they focused on OXT-based interventions for ASD) or because they were not full-text publications (i.e., letters and book chapters). Out of the resulting 134 articles, 119 were excluded from further analysis because they failed to meet the aforementioned inclusion criteria. Finally, 11 publications remained for the full-text analysis, with an additional four obtained through bibliography screening. The detailed selection procedure is presented in **Figure 3**. The outcomes of the analysis of selected publications among those included to the review are shown in **Tables 1**–**3**.

**Table 1:** A detailed analysis of selected publications regarding the single-nucleotide polymorphism (SNP) association in genes oxytocin receptor (OXTR) and arginine vasopressin 1A receptor gene (AVPR1a) and 1b in the pathogenesis of autism spectrum disorder (ASD).

No.	Publication	Type/data	Results
Studies regarding genes for *OXTR*			
1.	Wu et al., 2005 (29)	• 195 children with ASD (Chinese population) and their parents • 174 boys and 21 girls aged 6.7 ± 2.9 years	It was shown that some alleles of polymorphisms within the OXTR gene may be a risk factor for ASD. In particular, those located in the promoter region may significantly alter the level of gene expression and therefore may have an effect on their function.
2.	Jacob et al. 2007 (30)	• A group of 57 Caucasian children with ASD and their parents; 45 boys and 12 girls; mean age 6.4 ± 3.5 years	A significant association was detected of the “G” allele of the polymorphism rs2254298 with ASD development (p = 0.03). No such association was observed for rs53576.
3.	Lerer et al., 2008 (31)	• 152 children (Israeli population) diagnosed with ASD; 128 boys and 24 girls; aged 2 to 33 years	For two polymorphisms—rs2268494 (A/T) intron 3 and rs1042778 (G/T) exon 4—a significant association with the risk of ASD development in children was observed. The analysis of haplotypes (2 to 8 polymorphisms) revealed that in most cases, they manifest much stronger, significant associations with both ASD and the total score for VABS and IQ. It is worth noting that in each statistically significant haplotype, the “G” allele of rs2254298 was included.
4.	Liu et al., 2010 (32)	• A study on a group of 282 children diagnosed with ASD (Japanese population); 246 boy and 42 girls; mean age 21.75 years • Control group of 440 nonrelated healthy individuals; 272 men and 168 women; mean age 40.9 years.	In the ASD group, a statistically significantly higher frequency of the following polymorphisms was observed: rs237887 allele “A” (p = 0.023); rs2268491 allele “T” (p = 0.004); rs2254298 allele “A” (p = 0.001); and rs2268495 allele “A” (p = 0.032). The frequency of rs53576 (the “G” allele) was on the verge of statistical significance (p = 0.053). The “A” allele of rs2254298 was strongly associated with the risk of ASD (the frequency in the control group: 0.25; in the studied group: 0.33). The analysis of the obtained results indicates a possible significant participation of the OXTR gene in the development of ASD in the Japanese population.
5.	Wermter et al., 2010 (33)	• A study on 100 children with autism; 95 boys and 5 girls; age 6–24 years, mean 12.2 ± 4.7 years	Only the “C” allele of polymorphism rs2270465 showed a statistically significant association with ASD (p = 0.01). In the haplotype association analysis (up to four variations), the strongest evidence for the association with ASD was observed for the following variation: rs237851–rs6791619–rs53576–rs237884 (T–G–T–T); p = 0.007. In the analysis of up to six variations, the strongest factor was the abovementioned haplotype in combination with any 2 out of 11 other polymorphisms, which create significant models with it.
6.	Parker et al., 2014 (34)	• 79 children diagnosed with ASD (47 with classic autism and 32 with PDD-NOS); 17 girls and 62 boys; 52 siblings of the studied group with excluded diagnosis of ASD; 23 girls and 29 boys; 62 children in a neurotypical control group, including 22 girls and 40 boys	No direct statistically significant effect was observed of polymorphisms within the OXTR gene and the OXT concentration on the ASD phenotype development. A statistically significant, strongly additive effect was observed of selected alleles of polymorphisms within OXTR and the OXT concentration on the development of social cognition impairments in all groups included in the analysis. A significant impairment was observed in affect recognition for carriers of the “G” allele of rs53576 in the OXTR gene compared with carriers of the “A” allele. Carriers of the “A” allele of rs2254298 of the OXTR gene presented significantly worse results in terms of global social functioning as measured by the SRS total score. The outcomes of the Autism Diagnostic Interview-Revised score (A1) ADI-R were significantly worse for the carriers of the “A” allele of rs2254298 of OXTR. This suggests that the analyzed polymorphisms of the OXTR gene and the oxytocin concentration are not a risk factor for ASD. Second, these features do not differentiate between ASD and neurotypical patients. Third, they have a significant effect on cognitive deficits.
7.	Harrison et al., 2015 (35)	• The study is based on patients and data of the Simons Simplex Collection [Simons Foundation Autism Research Initiative (SFARI)] database with an additional study of selected families • 1,061 children with ASD (16% girls and 84% boys), mean age 7.57; 65% of children represented the Caucasian race	The regression analysis showed statistically significant links between the outcomes in SRS subscales and 3 out of 11 SNPs within OXTR (p < 0.05): • The “A” allele of rs237884 (F(1.542) = 8.16; p = 0.004). • The “G” allele of rs7632287 was linked with worse total SRS scores (F(1.542) = 6.51; p = 0.01). • The “T” allele of rs237888 was linked with impaired joint attention measured by means of appropriate ADOS domains (F(1.977) = 4.45; p = 0.04). • The remaining SNPs did not exhibit any statistically significant dependencies with the performed questionnaire studies. • The outcomes of this study suggest that social interaction impairments and—to some degree—also repetitive behavior are associated with polymorphism within 3′UTR of the OXTR gene, which may lead to decreased expression of oxytocin receptor.
8.	Nyffeler et al., 2014 (36)	• 76 children with ASD (64 boys and 12 girls), 78 siblings (33 boys and 45 girls) and 99 neurotypical children (77 boys and 22 girls) as controls, age between 5 and 17 years	• No statistically significant differences were observed in the frequency of consecutive alleles of the studied polymorphisms in ASD patients compared with their siblings and healthy control.
9.	Baribeau et al., 2017 (37)	• 341 children with ASD [73 girls (21%)], mean age 10.6 years; 276 children with attention deficit hyperactivity disorder (ADHD) [61 girls (22%)], mean age 10.3 years. 62.8% of ASD population was Caucasian; 69.9% of ADHD population was Caucasian	• rs53576 “G” allele was significantly correlated with higher outcome of SCQ score across both groups. • In ADHD group, carriers of “G” allele of rs53576 presented significantly worse outcome in RMET test; no such reliance was found in ASD group. • rs2254298 “A” allele was significantly correlated with higher outcome of SCQ score across both groups. • There was no significant link between rs237887 and SCQ and RMET scores in either group. • There was no significant link between rs13316193 and SCQ and RMET scores in either group.
Studies regarding genes for OXTR and AVPR			
1.	Francis et al., 2016 (38)	• 210 children diagnosed with ASD (age = 10.23 ± 6.25 years; 37 girls and 173 boys)	• SNPs within AVPR1b statistically significantly associated with the risk of ASD diagnosis: rs35369693 (G) (p = 0.025) rs28632197 (A) (p = 0.006) Detailed analyses of relationships between domains of ADOS and ADI-R scores did not achieve statistical significance. • SNP within OXTR statistically significantly associated with the risk of ASD diagnosis: rs2268493 (T) (p = 0.05) SNPs within OXTR statistically significantly associated with ASD symptoms: rs2268493 (ABC domain of social withdrawal, p = 0.013; ADI-R limited behavior patterns, p = 0.039) rs4686302 (ABC domain of social withdrawal, p = 0.036; ABC domain of stereotypy, p = 0.018) rs2254298 (A) (ADOS domain of social affect, p = 0.023). • Also, haplotypes composed of polymorphisms rs53576–rs2254298–rs2268493 were analyzed. Statistical significance was obtained in association with the following: ASD: rs2254298–rs2268493 A–T, p = 0.026 ADOS-social affect: rs53576–rs2254298–rs2268493 G–A–T, p = 0.08 rs53576–rs2254298 G–A, p = 0.017 rs2254298–rs2268493 A–T, p = 0.016 ABC-social withdrawal: rs53576–rs2254298–rs2268493 G–G–C, p = 0.009 rs53576–rs2268493 G–C, p = 0.009 rs2254298–rs2268493 A–T, p = 0.036 • Microsatellites AVPR1b did not reveal any statistically significant associations with the risk of ASD or its symptoms.
Studies regarding genes for AVPR1a			
1.	Wassink et al., 2004 (39)	• 125 children with ASD recruited in the USA	No statistically significant polymorphisms within AVPR1a were found in the analyzed group of patients, which could be the basis for deficits observed in the course of ASD. However, as the authors themselves admit, they were searching for specific variants within genetic code, the lack of which does not exclude the presence of other variants within the AVPR1a gene in this group of patients.
2.	Yirmiya et al., 2006 (40)	• A group of 128 children with ASD (125 boys and 3 girls); age between 2.08 and 33.66 years	No significant association was observed between ASD and RS1 and RS3. There was a significant association with AVR. A statistically significant association was found for seven haplotypes, which were variants of the studied polymorphisms. The outcomes also suggest that there is a significant participation of AVPR1a in the functioning of the “social brain.”
3.	Yang et al., 2017 (41)	• 151 Korean children diagnosed with ASD; age 79.9 ± 35.6 months; 86.1% boys and 13.9% girls	A statistically significant association was observed between ASD and the following polymorphisms: rs7294536 “A” allele χ^2^ = 9.328, p = 0.002 rs10877969 “A” allele χ^2^ = 11.52, p < 0.001 It was also observed that the “G” allele of rs7294536 may serve a protective nature by decreasing the severity of social interactions disorders. However, the precise mechanism requires further study. For all three studied polymorphisms, the analysis of pairs of alleles revealed a statistically significant association with ASD for the A/A/A form (Z = 3.26; p = 0.001) and G/A/G (Z = −3.03; p = 0.002). Also, for rs7294536, an association was observed between genotype A/A (as opposed to genotypes A/G and G/G) and worse results of ADI-R score within the subscale of peer relationships (p = 0.04). The study points to a possible association between polymorphisms within rs7294536 and rs10877969 and the development of ASD. However, it should be remembered that these variations are located within the promoter region of AVPR1a, and they couple with polymorphism within RS1 and RS3 whose association with ASD was observed by other authors. Consequently, they might not have a direct association with ASD, yet they may suggest the presence of polymorphism RS1 or RS3.
4.	Tansey et al., 2011 (42)	• 177 Caucasian children with ASD of the Irish origin; the boy/girl ratio was 4.68:1 • Studied polymorphisms: • rs3803107, rs1042615, rs3741865, rs11174815, and microsatellites: • RS3, RS1, and AVP	Out of all the analyzed SNPs, only the “A” allele in the polymorphism rs11174815 showed a statistically significant association with ASD at p = 0.008. As for RS1, also a weak association of the short allele with ASD was observed OR = 1.44 (95% CI: 1.02 to 2.04), p = 0.036. The longer allele of RS1 showed 2.7 times higher relative activity than the short-repeat allele (p = 0.0005). Similarly, the long allele of RS1 was 1.4 times more active than the short-repeat allele (p = 0.0081). Short-repeat alleles of RS1 and RS3 in the group of ASD children were inherited more often than in the general population. The outcomes of the study show that there is a possible cause-and-effect association between the presence of the RS1 and RS3 polymorphisms and a lower expression of the AVPR1a gene, which may be responsible for the development of social cognition deficits in ASD. Interestingly, the association between the microsatellites and ASD was only observed for RS1, and it was weak.
5.	Kantojärvi et al., 2015 (43)	• 224 children with ASD (49 girls and 175 boys); girls’ mean age 12 years; boys’ mean age 16 years	There was a statistically significant association with the development of the ASD phenotype for the variant of the microsatellite RS1 of the length of 310 base pairs and the “A” allele of the polymorphism rs7307997.

CARS, Childhood Autism Rating Scale; SRS, Social Responsiveness Scale; VABS-2, Vineland Adaptive Behavior Scales, Second Edition; RBS, Repetitive Behavior Scale; ABC, Autism Behavior Checklist; SCQ, Social Communication Questionnaire; RMET, Reading Mind in the Eyes Test; PDD-NOS, Pervasive Developmental Disorder-Not Otherwise specified; OR, odds ratio.

**Table 2:** Review of gene polymorphisms for OXTR (mentioned by at least two authors), which revealed a statistically significant association with ASD and the suggested alleles that increased risk of ASD.

	rs2254298	rs53576	rs2268494	rs1042778	rs2268495	rs2268491	rs237887	rs7632287	rs237888	rs2268493
Wu et al., 2005 (29)	A: SS	A: SS								
Jacob et al., 2007 (30)	G: SS	N/S								
Jacob et al., 2007 (30)	G: SS	N/S								
Lerer et al., 2008 (31)	N/S	N/S	A: SS	G: SS						
Liu et al., 2010 (32)	A: SS	G: SS			A: SS	T: SS	A: SS			
Wermter et al., 2010 (33)	N/S	N/S			N/S					
Tansey et al., 2010 (44)			N/S	N/S	N/S			G: SS	N/S	
Campbell et al., 2011 (45)	N/S	N/S		G: SS			N/S	G: SS	N/S	T: SS
Parker et al., 2014 (34)	A: SS	G: SS								
Nyffeler et al., 2014 (36)	N/S	N/S	N/S							
Napoli et al., 2014 (46)	N/S	N/S								T: SS
Harrison et al., 2015 (35)		N/S		N/S	N/S	N/S		G: SS	T: SS	
Francis et al., 2016 (38)	A: SS	N/S								T: SS
Shuhan et al., 2017 (47)	N/S									
Ribeiro et al., 2018 (48)				G: SS						
LoParo & Waldman, 2015* (25)	A: SS					T: SS	A: SS	A: SS		
Social cognition scales that were associated with selected SNPs across literature	Allele (A): VABS CS; SRS; ADI-R A1; ADOS SA; RMET; SCQ	Allele (G): NEPSY AR; RMET; SCQ	No significant associations	No significant associations	No significant associations	No significant associations	Allele (A): SCQ; RMET	Allele (A): SRS; ADOS SA	Allele (C): VABS SS; Allele (T): SRS	No significant associations

SS, statistically significant association; N/S, not statically significant association; VABS CS, Vineland Autism Behavior Scale Communication Subdomain; VABS SS, Vineland Autism Behavior Scale Socialization Skills; SRS, total score of Social Responsiveness Scale; ADI-R A1, Autism Diagnostic Interview-Revised domain A1, “failure to use nonverbal behaviors to regulate social interaction”; ADOS SA, Autism Diagnostic and Observation Schedule Social Affect; RMET, Reading Mind in the Eyes Test; SCQ: Social Communication Questionnaires; NEPSY AR, A Developmental NEuroPSYchological Assessment, Affect Recognition. (Allele (X), which allele is associated with listed scales).

**Table 3:** Review of gene polymorphisms for AVPR1a (mentioned by at least two authors), which revealed a statistically significant association with ASD and the suggested alleles that increase the risk of ASD.

	RS1	RS3	rs7294536	rs10877969	rs11174815	rs7307997
Wassink et al., 2004 (39)	N/S	N/S				
Yirmiya et al., 2006 (40)	N/S	N/S				
Yang et al., 2017 (41)			A: SS	A: SS		
Tansey et al., 2011 (42)	SS	N/S			A: SS	
Kantojärvi et al., 2015 (43)	SS	N/S				A: SS
Social cognition scales that were associated with selected SNPs across literature	No significant associations	No significant associations	Allele (G): SCQ; SRS; ADI-R; VABS	Allele (G): SCQ; SRS; ADI-R	No significant associations	No significant associations

The most noteworthy links between SNPs in OXTR and SC were found for SNPs rs2254298, rs53576, and rs7632287.

SNP rs2254298 was mentioned in the majority of included studies, and in most studies, it was associated with a diagnosis of ASD. SC was linked to deficits in global social functioning as measured by Social Responsiveness Scale (SRS), outcome of the Social Communication Questionnaire (SCQ) score, deficits in reciprocal social interaction, and the outcome of the social affect domain of *Autism Diagnostic Observation Schedule*, Second Edition (ADOS-2).

In the majority of studies, SNP rs53576 was not associated with ASD itself; however, it was significantly linked to, among others, deficits in social affect recognition, SCQ total score, and social withdrawal (especially as a part of haplotype with rs2254298 and rs2268493) in this group of patients. Furthermore, some authors pointed out that this might be associated with SC deficits across both the ASD and neurotypical populations.

SNP rs7632287 was not only significantly associated with ASD across all populations studied but was also significantly associated with SC domains of ADOS and Autism Diagnostic Interview-Revised (ADI-R), as well as with the total score and individual domains of SRS-2.

Significant associations were also found for rs4686302 and rs2268493 and the presence of social withdrawal, rs237884 for impaired global social functioning, and rs237888 for deficits in joint attention.

As far as SNPs in the AVPR1a gene, rs7294536 is strongly associated with deficits in social interactions, SRS and SCQ total scores, and correlation with ADI-R peer relationships subscale. Similarly, rs10877969 was significantly associated with SCQ, SRS total scores, social domains of ADI-R, and social domains of Vineland Autism Behavior Scale. The other SNPs in AVPR, although pointed out as ASD risk factors, were not significantly associated with SC deficits.

## Discussion

One of the first observed genetic abnormalities in ASD patients was a reduction of the size of the 0.7 Mbp covering the region of chromosome 3, where the OXTR gene is located (29, 31). This discovery initiated studies on its association with ASD, especially in the context of SC dysfunctions. The analysis of reports on this subject seems to suggest that, indeed, an association of this kind exists.

This is especially clear for the “A” allele of SNP rs2254298 for which the association with ASD and SC dysfunctions in the course of ASD revealed a statistical significance in most studies. Interestingly, in the meta-analysis carried out by LoParo and Waldman in 2015 (25), the OR for the “A” allele of rs2254298 equaled 1.15 (95% CI: 0.93 to 1.43; *p* = 0.0038), which could imply a lack of significant influence on the risk of development of ASD between the alleles of this polymorphism. However, as LoParo and Waldman stressed, among the analyzed studies, there was a significant heterogeneity (*Q*: χ^2^ = 11.2, *p* = 0.048, *I* ^2^ = 55%), most likely caused by considerable differences in sex proportions, ethnic groups, or the age of the individuals recruited to the studied groups. In the analysis of associations between the scales of social functioning and rs2254298, the results seem to be more coherent. In 2014, Parker et al. (34) showed a significant association of this SNP with the total SRS score as well as with the A1 domain of the ADI-R. In 2016, Francis et al. (38) observed that rs2254298 was not only itself significantly associated with the ADOS domain of social affect (the “A” allele; *p* = 0.023), but also its alleles constituted the element of all haplotypes, revealing a significant association with social dysfunctions measured with the ADOS social affect domain and the ABC questionnaire.

Despite numerous studies that analyzed the potential role of the alleles of SNP rs53576 in the course of ASD, the association did not present any statistical significance in most of the reports. Instead, it seems that rs53576 modulates the SC in a significant way in all the studied groups—among both clinical and neurotypical individuals (49, 50). Its alleles may also constitute a significant element of haplotypes that exhibit a significant association with ASD. In the 2014 study of Slane et al. (51), the authors observed that there are much worse SRS-2 scores and Child Behavior Checklist scores in the presence of the haplotype composed of the alleles “G” rs53576 and “A” rs2254298 than in individuals with the haplotype combined of alleles “A” and “G,” respectively. Although the study of Parker et al. in 2014 showed no significant associations with the SRS-2 scores for the polymorphism rs53576, the carriers of the “G” allele revealed significantly worse results in their ability to interpret their effect in others (34).

OXTR SNP rs7632287 is yet another interesting polymorphism whose association with ASD obtained a statistical significance in all studied populations. The “G” allele rs7632287 showed a statistically significant association with worsening of SRS scores, especially regarding SC such as in the 2015 study of Harrison et al. (35). In a study carried out by Campbell et al. in 2011 (45), there was a statistically significant association with the SC domains of ADOS (*p* < 0.004) and ADI-R (*p* < 0.033), as well as with the total score and individual domains of SRS-2 (*p* = 0.029). A significant association with individual SRS-2 domains was also observed for the “T” allele of SNP rs2268493 in their study.

In 2016, these results were replicated by the team of Francis et al. (38) who proved a further association of the “T” allele of rs2268493 with the social withdrawal domain of the ABC. Interestingly, the variations of alleles of rs2268493, rs2254298, and rs53576 were the only haplotypes to be statistically significantly associated with worse outcomes in the scope of social domains of ADOS and ABC in this study. However, due to limited literature regarding these and the remaining polymorphisms presented in **Table 2**, further research is necessary to draw unequivocal conclusions. In 2008, Lerer et al. (31) also paid attention to the significance of research on the haplotypes of the alleles of the analyzed polymorphisms. In a population of 152 children with ASD, Lerer and colleagues showed that the alleles of rs2254298 were present in all the haplotypes, revealing a statistically significant association with an ASD diagnosis despite the fact that none of the alleles of the SNP rs2254298 themselves revealed a significant association with ASD individually. Similar results were also obtained by Slane et al. in 2014 (51), who observed that the variation of the alleles “A” rs2254298 and “G” rs53576 was more strongly associated with ASD than each of the SNPs.

The database of reports for the arginine vasopressin receptor was considerably poorer—and just as in the case of OXTR—equally heterogeneous and often contradictory. The greatest number of reports refers to microsatellite polymorphisms within the RS1 and RS3 microsatellites of the AVPR1a gene. The majority of available reports did not show a statistical significance for the association between RS3 and ASD, although in the study by Yirmiya et al., haplotypes including RS1, RS3, and AVR microsatellites were significantly linked to the risk of ASD, and furthermore, RS3 itself was associated with the outcome of ADOS-G. In the case of RS1, the opinions were divided between the confirming reports of Kantojärvi et al. in 2015 and Tansey et al. in 2011 and the negating studies by Wassink et al. in 2014 and Yirmiya et al. in 2006 (39, 40, 42, 43). As for SNP, in 2017, Yang et al. (41) showed a significant association for the “A” alleles of rs7294536 and rs10877969. The “A” allele of rs7294536 also showed a significant association with worse social functioning measured with ADI-R. However, literature on the subject of SNPs in the AVPR1a/1b genes is extremely poor, and therefore, further research is required to confirm or reject the hypothesis of their association with ASD and SC dysfunctions in the course of ASD.

The analysis of available literature seems to indicate that there is an association between ASD and SC dysfunctions in the course of ASD and selected alleles of polymorphisms within OXTR and AVPR1a. However, previous studies neither specify the nature of this association in an unequivocal way nor select genotypes that are the basis for this association. SC is a highly complex process requiring a vast regulatory network involving genetic, epigenetic, and environmental factors, and therefore, slight dysfunctions in different parts of it may consecutively lead to profound changes in functioning of each affected individual (52). Therefore, although frequent across the ASD population, different polymorphisms within OXTR and AVPR1a genes might not be present in all cases of ASD, which may explain the observed heterogeneity of outcomes across studies. Furthermore, polymorphisms within OXTR and AVPR genes might be responsible for SC deficits, regardless of ASD diagnosis. This was indicated, among others, by the outcomes of a study by Parker et al. published in 2014 (34), in which the authors conclude that although all studied SNPs were linked to SC deficits across both the study and control groups, they were not associated with ASD itself. Therefore, the presence of SNPs in OXTR and AVPR genes may exacerbate SC deficits in patients with ASD but might not be a condition sine qua non for their development. On the other hand, this may explain contradictory results in literature concerning the association between OXTR and AVPR1a genes and the risk of ASD (10), as well as the lack of such information in genome-wide association studies of ASD populations (53, 54). Furthermore, while looking for associations between SNPs and specific groups of symptoms, the risk of linkage disequilibrium (LD) must be taken into account. LD is the non-random association of alleles of different loci that occur together at a rate higher than expected by chance alone (55). Therefore, certain alleles of selected SNPs might seem to be linked to SD in the course of ASD. Nevertheless, there is no real connection, and in fact, it is associated with other functional SNPs that are responsible for establishing a significant link to SD. For instance, in a study by Jacob et al. (30), the authors highlight the difference between Caucasian population (G allele) and Chinese Han population (A allele) in allele of SNP rs2254298, which seems to be linked to ASD vulnerability. This variation indicates that there might be no direct difference between both alleles in their influence on ASD vulnerability and therefore no direct link between ASD vulnerability and SNP rs2254298 itself. Instead, they might be in LD with another yet unspecified susceptibility variant in OXTR or other gene, and difference in significant alleles between studied populations might be attributable to the ethnic variations in LD. A more detailed analysis of LD in the region of OXTR was presented inter alia in a publication by Harrison et al. (35).

Moreover, these findings suggest that it might be worthwhile to consider the existence of two subtypes of ASD based on their associations with OXTR and AVPR genes. Distinguishing between these subtypes and developing some cost-effective tools to facilitate their diagnosis in a clinical setting may pave the road for an emergence of new treatment methods or increase efficacy of already existing ones, such as intranasal administration of OXT. However, to achieve this goal, further well-designed studies on associations between OXTR, AVPR, and SC in ASD are required, which must take into account a few crucial issues. First and foremost, previous studies (31, 51) suggest that the assessment of single polymorphisms may in fact never bring unequivocal results. Future research studies should focus on the simultaneous analysis of the widest possible range of SNPs in both genes, taking into consideration their interactions. Second, it should be remembered that most papers published so far have covered mixed groups of boys and girls, without dividing children into separate sexes. Yet it has been postulated that there is a sexual dimorphism in terms of the role played by AVP and OXT in the CNS (56–58). Consequently, this means that gender may constitute a strong disruptive factor that makes it impossible to draw reliable conclusions on the basis of obtained results. Similarly, it should also be taken into consideration that there are possible differences within the frequency of studied polymorphisms depending on ethnicity (30–32), which may also be the reason for the observed heterogeneity of the available literature. Finally, while interpreting findings on associations of SNPs with specific groups of symptoms, such as SC deficits, the risk of LD between each of the SNPs must be taken into account. Further research into this field, which will maintain a more uniform structure of studied groups, is definitely necessary, and in the future, it may help bring a better understanding of the pathogenesis of SC dysfunctions and their relation to ASD.

## Author Contributions

KW, AS, MJ-K: Conceived the presented idea, planned the methodology, performed the review, interpreted literature, wrote the paper, edited, and reviewed manuscript.

## Conflict of Interest Statement

The authors declare that the research was conducted in the absence of any commercial or financial relationships that could be construed as a potential conflict of interest.
